# Interactions between Amyloid-Β Proteins and Human Brain Pericytes: Implications for the Pathobiology of Alzheimer’s Disease

**DOI:** 10.3390/jcm9051490

**Published:** 2020-05-15

**Authors:** Donald J. Alcendor

**Affiliations:** Center for AIDS Health Disparities Research, Department of Microbiology, Immunology and Physiology, School of Medicine, Meharry Medical College, Nashville, TN 37208, USA; dalcendor@mmc.edu

**Keywords:** Alzheimer’s disease, pericytes, β-amyloid, blood–brain barrier, neurovascular unit, neurodegeneration, neuroinflammation, retinopathy

## Abstract

Alzheimer’s disease (AD) is a progressive neurodegenerative disease that is the most common cause of dementia, especially among aging populations. Despite advances in AD research, the underlying cause and the discovery of disease-modifying treatments have remained elusive. Two key features of AD pathology are the aberrant deposition of amyloid beta (amyloid-β or Aβ) proteins in the brain parenchyma and Aβ toxicity in brain pericytes of the neurovascular unit/blood–brain barrier (NVU/BBB). This toxicity induces oxidative stress in pericytes and leads to capillary constriction. The interaction between pericytes and Aβ proteins results in the release of endothelin-1 in the pericytes. Endothelin-1 interacts with ET_A_ receptors to cause pericyte contraction. This pericyte-mediated constriction of brain capillaries can cause chronic hypoperfusion of the brain microvasculature, subsequently leading to the neurodegeneration and cognitive decline observed in AD patients. The interaction between Aβ proteins and brain pericytes is largely unknown and requires further investigation. This review provides an updated overview of the interaction between Aβ proteins with pericytes, one the most significant and often forgotten cellular components of the BBB and the inner blood–retinal barrier (IBRB). The IBRB has been shown to be a window into the central nervous system (CNS) that could allow the early diagnosis of AD pathology in the brain and the BBB using modern photonic imaging systems such as optical coherence tomography (OCT) and two-photon microscopy. In this review, I explore the regulation of Aβ proteins in the brain parenchyma, their role in AD pathobiology, and their association with pericyte function. This review discusses Aβ proteins and pericytes in the ocular compartment of AD patients as well as strategies to rescue or protect pericytes from the effects of Aβ proteins, or to replace them with healthy cells.

## 1. Introduction

Alzheimer’s disease (AD) is a pervasive and progressive neurodegenerative disorder that is expected to affect 14 million people in the US by 2050, with increasing incidence in aging populations. Currently, there are no curative treatments for AD [1,2]. While existing therapies can address the symptoms of AD, they cannot inhibit disease progression. In fact, they only offer modest improvements in cognition and overall functioning. AD pathology has largely been attributed to the progressive accumulation of extracellular amyloid-β (Aβ) proteins in the brain parenchyma, subsequently resulting in the formation of senile plaques and neurofibrillary tangles composed of hyperphosphorylated tau proteins [3,4]. These plaques also occur in vessels, causing cerebral amyloid angiopathy [4,5,6,7]. Over time, these phenomena result in neuronal damage that coincides with loss of synapses and synaptic plasticity, leading to progressive cognitive impairment and dementia.

Epidemiologic studies have uncovered a vascular component in the development of AD [8]. One of the most prominent initial changes in AD pathology is a decrease in cerebral blood flow (CBF) [8]. Since vascular resistance in the brain parenchyma is associated with capillaries, cerebral pericytes may play a role in the development of AD [9]. The human blood–brain barrier (BBB) consists of brain microvascular endothelial cells, brain pericytes, and astrocytes; together, they form the neurovascular unit (NVU) [10,11,12]. Pericytes of the BBB play an essential role in several microvascular functions, including angiogenesis, vascular remodeling, and the regulation of microcirculation, as well as the generation and maintenance of the BBB [13,14,15,16,17]. Pericyte dysfunction has been observed in AD pathology [18,19,20,21]. Aβ proteins induce neurovascular dysfunction, which leads to functional changes in the brain microvasculature, subsequently impairing capillaries in their response to neuronal activity [22,23]. Using human brain slices and a mouse model of AD, Nortley et al. showed that oxidative stress caused by Aβ toxicity leads to capillary constriction via the generation of reactive oxygen species (ROS) by reduced nicotinamide adenine dinucleotide phosphate oxidase 4 (NOX4) [24]. These ROS then trigger the release of endothelin-1. Endothelin-1 interacts with ET_A_ receptors to cause pericyte contraction and subsequent capillary constriction [24]. Interestingly, the authors did not observe pericyte constriction in areas of the mouse brains with no Aβ deposition [24]. Findings from this study suggest that brain vascular pericytes are the link between Aβ proteins and vascular dysfunction in AD [24].

The underlying mechanisms that support the role of pericytes in AD pathobiology are unclear. The lack of knowledge in this regard constitutes an important information gap in our understanding of the interactions among cellular components of the NVU and Aβ proteins in the development of AD. Since brain pericyte dysfunction leads to dysfunction of the NVU/BBB, pericytes play a major role in the predisposition and development of AD. The Aβ_40_ and Aβ_42_ alloforms exist in amyloid deposits in the brains of AD patients and in cerebral pericytes [25,26]. Perivascular drainage of Aβ peptides that represent insoluble deposits in the brain parenchyma and in blood vessel walls that can lead to cerebral amyloid angiopathy (CAA) is essential [27]. More specifically, CAA involves the pathologic deposition of Aβ within cortical and leptomeningeal arteries, arterioles, capillaries and, in rare cases, the venules of the brain. Dysfunction of perivascular drainage pathways to eliminate Aβ in interstitial fluid (ISF) from the brain can result in intracerebral hemorrhage due to the rupture of Aβ-laden arteries and cerebral vascular dysfunctions seen in AD patients, leading to neuronal injury, cognitive decline, and dementia [27]. The BBB is the primary route of clearance for Aβ_40_ and Aβ_42_ [28]. Aβ proteins have been implicated in pericyte loss and dysfunction, leading to the BBB impairment that contributes to neurodegeneration [18,19,20,21]. A study in mice showed that Aβ_40_ clearance via the BBB is mediated by scavenger receptor low-density lipoprotein receptor-related protein-1 (LRP-1) [29]. Pericyte degeneration or loss as well as LRP-1 downregulation are predominant mechanisms that compromise the BBB in AD patients and AD animal models [30,31].

The understanding of functional changes in brain pericytes in AD patients and the mechanisms associated with AD pathobiology may lead to strategies for protecting and/or preserving pericyte function. These strategies could serve as early intervention and/or treatment options that may delay, prevent, or limit AD progression. These options may also be applicable to other conditions that affect the BBB such as multiple sclerosis, strokes, and various neurological tumors including glioblastoma multiforme.

## 2. Aβ Proteins and Pericytes

Coined by Virchow, the word “amyloid” was derived from the Latin word *amylum* and the Greek word *amylon* for starch that stained positive with iodine [32,33]. Friedrich and Kekule later correctly identified amyloid to consist mainly of protein and a small fraction of glycosaminoglycans [34]. All known types of AD (early-onset, late-onset, and familial) seem to involve the aberrant accumulation and/or dysfunctional clearance of Aβ proteins. Amyloidosis refers to the aberrant accumulation and deposition of amyloid fibrils in the central nervous system (CNS) that are associated with several age-related human pathologies, including AD and Parkinson’s disease. Nearly 50 unique proteins or peptides have been associated with the amyloid fibrils that exist in amyloid-based diseases [35,36]. These amyloid fibrils self-assemble and accumulate extracellularly as plaques and intracellularly as intranuclear inclusions. Nearly 40 years of research on amyloid accumulation and deposition have resulted in technologies such as cyro-electron microscopy and solid-state NMR spectroscopy that yielded the first near-atomic-resolution structures of amyloid fibrils formed in vitro [37]. Conformation-dependent antibodies have been used to separate Aβ proteins into different structural classes [38,39,40] that may play different pathological roles in AD. These structural classes include fibrillar and prefibrillar oligomers. Aβ_40_ fibrillar oligomers are small aggregates that have a high β-sheet content and are structurally similar to fibrils, with some disruptions in the β-sheet stacking [41]. Breydo et al. examined structural differences among fibrillar oligomers, prefibrillar oligomers, and fibrils using Raman, Fourier transform infrared spectroscopy (FTIR), and Circular dichroism (CD) spectroscopy. They found that fibrillar oligomers, although less stable, are structurally similar to fibrils, while prefibrillar oligomers are much less ordered [42]. These findings support previously proposed models of Aβ oligomers.

The variable organization of α-helices and β-strands in globular proteins associated with Aβ proteins consists of more than 1375 distinct fold patterns [43]. This phenomenon leads to different clinical presentations involving the aggregation of the same protein, which may explain why amyloid-based diseases are so difficult to understand and treat [43]. Since the first documented case of AD in a patient by Alois Alzheimer in 1907, more than 50 disease-causing amyloidogenic proteins have been identified [44,45]. There are a multitude of genetic variants that predispose individuals to amyloid-based diseases. For instance, individuals with trisomy 21, which contains the gene encoding amyloid precursor protein (APP) from which Aβ_40/42_ are derived, are at a higher risk of AD [46]. What initiates the onset of amyloid-based diseases remains unclear. Moreover, strategies that decrease the concentration of amyloidogenic monomers to reduce amyloid load [47] or promote the sequestration of monomers into non-amyloid amorphous aggregates [48] could have deleterious effects on the normal function of these monomers. Therefore, an understanding the molecular basis for amyloid aggregation and deposition will aid the development of novel therapeutic strategies for combating amyloid-based diseases like AD.

**Figure 1:**
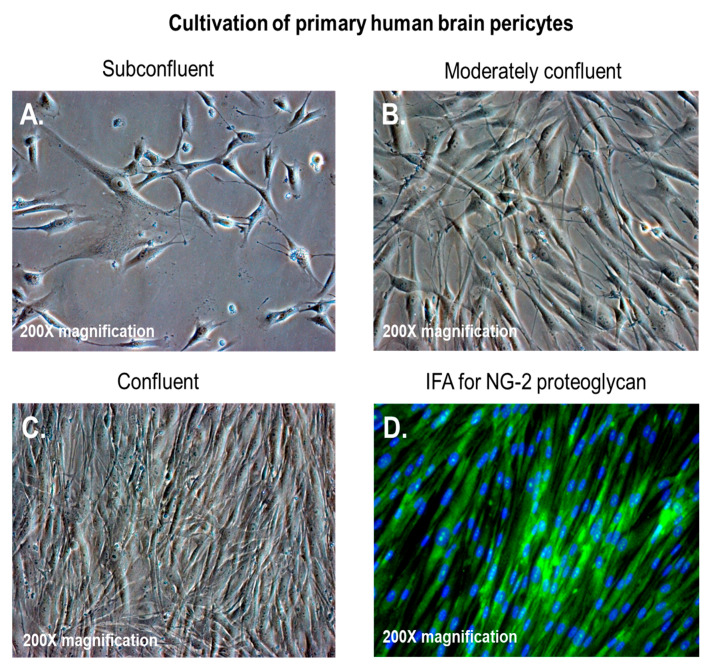
Brain vascular pericytes in vitro. (**A**–**C**) Primary human brain pericytes in culture, from low to high confluency; (**D**) primary human brain pericytes stained with a monoclonal antibody against neural/glial antigen-2 (NG2) proteoglycan. The total magnification is 200× ([17], Figure 1A,C).

**Figure 2:**
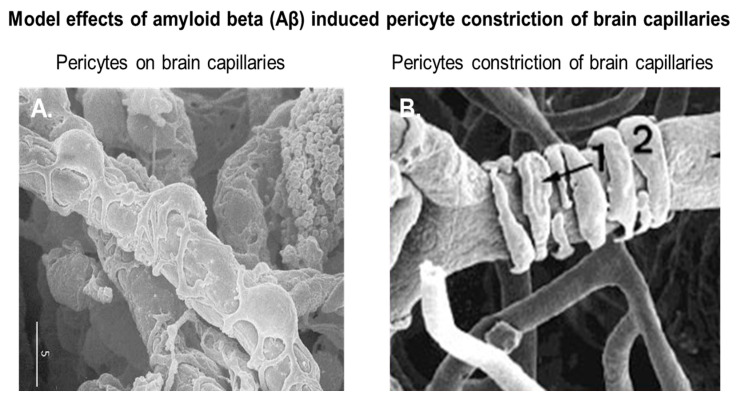
Brain vascular pericytes in vivo. (**A**) Pericytes on rat brain capillaries; (**B**) rat brain capillaries undergoing constriction by vascular pericytes. The marker measures 5 microns. Modified with permission from Pearson Education (unpublished data). (1) is an under configuration wrapping of pericyte extensions around the capillary; (2) is an over configuration wrapping of pericyte extensions around the capillary.

Pericytes, also known as Roget cells or mural cells, were first described by Eberth and Rouget in the 1870s. The name “pericyte” was introduced by Zimmermann in 1923 [49,50,51,52]. Pericytes are heterogeneous, tissue-specific, and multipotent cells that are abluminal to endothelial cells. They have contractile properties due to the expression of contractile and cytoskeletal proteins such as α-smooth muscle actin (α-SMA), vimentin, desmin, myosin, and nestin. These proteins enable pericytes to extend their cytoplasm to wrap around the endothelial cells lining pre-capillary arterioles and post-capillary venules throughout the body [49,50,51,52]. In culture, primary brain pericytes maintain their long cytoplasmic extensions and stain positive for neural/glial antigen-2 (NG2) (Figure 1). Although brain pericytes do not possess any unique antigenic biomarkers, they can be identified by their expression of platelet-derived growth factor receptor-β (PDGFRβ), α-SMA, CD13, NG2, CD146, and desmin [53]. NG2 and CD13 are also present on vascular smooth muscle cells (vSMCs) in large vessels [53]. Unlike the vasculature of peripheral tissues, the vasculature in the retina and brain is known to have the highest pericyte-to-endothelial cell ratio [54,55]. The proximity of pericytes to endothelial cells facilitate signaling that is essential for vessel formation, vessel maturation, pericyte recruitment, and pericyte coverage [55]. Endothelin-1 signaling can induce the arrangement of brain vascular pericytes on capillaries (Figure 2A) in order to constrict capillaries and regulate CBF (Figure 2B). PDGF-β secreted by endothelial cells recruits pericytes to blood vessels by binding to PDGFRβ on pericytes, causing receptor dimerization, autophosphorylation, and activation [56,57]. The human BBB consists of astrocytes, pericytes, neurons, and the extracellular matrix; together, they form the NVU [58]. Under physiological conditions in the CNS, pericytes regulate BBB integrity via the use of tight/adherens junctions and transcytosis across the BBB. Pericytes regulate angiogenesis, phagocytosis, the clearance of toxic metabolites from the CNS, and CBF by inducing changes in capillary diameter, neuroinflammation, and multipotent stem cell activity [59]. Proinflammatory responses at the BBB are also mediated by PDGFRβ signal transduction in pericytes [60]. Transcriptional regulation of chemokines that promote endothelial expression of monocyte chemoattractant protein-1 (MCP-1), nitric oxide (NO), IL-1, IL-6, IL-12, and tumor necrosis factor-α (TNF-α) can influence the transvascular trafficking of macrophages and leukocytes into the brain parenchyma [59].

## 3. The Interaction between Aβ Proteins and Brain Pericytes in AD Pathobiology

AD is an amyloid-β-based disease in which a disruption in the production and clearance of Aβ proteins in the brain leads to Aβ protein accumulation and associated pathology. The association between Aβ proteins and AD emerged when Aβ plaques were detected in the brains of AD patients (1892) and in proteinaceous fibrils (1968). The Aβ species in AD patients are known as Aβ_40_ and Aβ_42_. These proteins form insoluble, highly neurotoxic fibrils that make up the Aβ plaques of which Ab_42_ is the major component [61]. Areas in the brain parenchyma containing Aβ plaques display signs of neuroinflammation, as indicated by the presence of activated glial cells and significant neuronal loss. A study by Segillo et al. revealed significant pericyte loss in the brains of AD patients compared to those of age-matched non-demented controls [62]. In addition, the authors found that pericyte loss correlated with increased Aβ deposition in the brains of AD patients and those of mice with AD [19,62]. In vitro studies have shown reduced pericyte survival and the loss of NG2 proteoglycan after exposure to Aβ_42_, as well as Aβ_42_ oligomer fibrils, respectively [63,64,65]. Although Ab_40_ is less prone to aggregate and form plaques compared to Ab_42_, it has been shown to form deposits in the vessel wall in the development of cerebral amyloid angiopathy (CAA) [66]. In CAA, pericytes degenerate and are less likely to survive after exposure to Ab_40_ aggregates [67,68]. Most recently, Shultz et al. observed a reduced number of hippocampal NG2+ pericytes and an association between NG2+ pericyte numbers and Aβ_40_ levels in AD patients. The authors showed that the effects of Ab_40_ on NG2+ pericytes are aggregation dependent. Specifically, while exposure to Ab_40_ monomers increased pericyte viability and proliferation and reduced caspase 3/7 activity, exposure to fibril-enriched Ab_40_ reduced pericyte viability and proliferation and increased caspase 3/7 activity [69]. These findings suggest that the formation of Ab_40_ fibrils and pericyte coverage reduction at the BBB have a positive feedback relationship that may exacerbate parenchymal and vascular Aβ accumulation.

Studies in humans have shown that up to 50% of the Aβ proteins produced by neurons and other types of cells in the brain travel across the BBB to the blood [70]. Cellular components of the BBB, including pericytes, are responsible for Aβ degradation and clearance via BBB into the blood [71]. During Aβ protein transport, Low-density lipoprotein receptor-related protein 1 (LRP1), an apolipoprotein E (apoE) receptor [72,73], mediates the internalization of soluble Aβ proteins at the abluminal side of the BBB [74,75,76] and subsequent Aβ transcytosis across the BBB. Phosphatidylinositol-binding clathrin assembly protein (PICALM), as well as small GTPases Rab5 and Rab11, regulate Aβ transcytosis, which results in Aβ exocytosis across the luminal side of the BBB into the blood [77]. Pericytes at the BBB participate in the clearance of toxic molecules and proteins, including Aβ proteins that are also eliminated by perivascular drainage pathways, by which interstitial fluid (ISF) and solutes are cleared from the brain [77]. A recent study by Ma et al. using APPSw/0 mice showed that Aβ proteins can accumulate in brain pericytes, underscoring the role of brain pericytes in Aβ clearance at the BBB [78]. The authors also showed that pericytes internalize and clear Aβ aggregates via an LRP1/apoE-specific mechanism. These findings could aid the development of novel therapeutic strategies targeting the LRP1/apoE pathway in pericytes [78].

**Figure 3:**
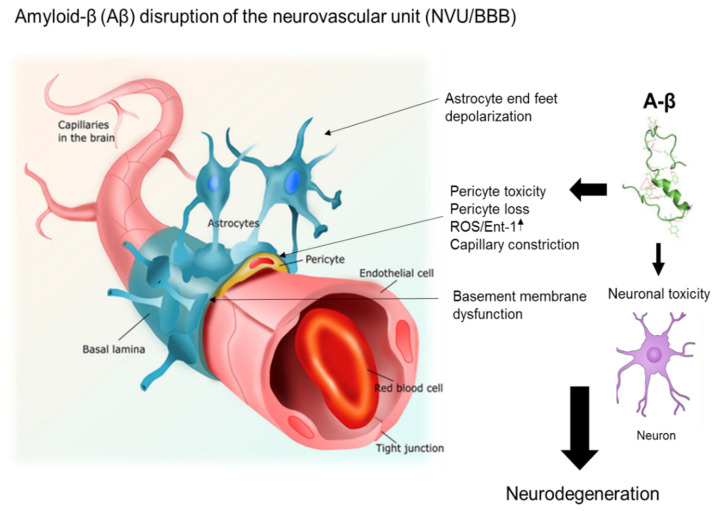
A model of the effects of amyloid beta (Aβ) proteins on pericyte function in the neurovascular unit (NVU). The effects of Aβ proteins on pericytes include pericyte loss, pericyte toxicity, induction of reactive oxygen species (ROS), and capillary constriction, all contributing to neuronal toxicity and neurodegeneration. Figure 3 was revised from previous studies by Alcendor [27]. For the original figure, I acknowledge Pearson Education Inc. (2014).

Aβ_34_ is a C-terminal-truncated isoform of Aβ that is an intermediate in the Aβ degradation pathway. Its production results from either matrix metalloprotease activity or from the degradation of Ab_40_ and Ab_42_ by γ-secretase [79]. The cleavage of Aβ_40/42_ by beta-site amyloid precursor protein-cleaving enzyme 1 (BACE1) also produces Aβ_34_ [80]. Braak staging of post-mortem hippocampal and cortical brain tissue from AD patients revealed that Ab_34_ immunoreactivity was confined to pericyte-associated brain capillaries in the early stages of AD [81]. This pericyte-associated Aβ_34_ immunoreactivity became largely undetectable at later stages of AD. In addition, using primary human brain pericytes, the authors of this study also observed a dose-dependent increase in Aβ_34_ levels upon exposure to recombinant Aβ_40_ peptides that correlated with a reduction in both Aβ_40_ uptake and BACE1 activity [82]. Finally, the authors proposed that Aβ_34_ is generated by a novel BACE1-mediated Aβ clearance pathway in the pericytes of brain capillaries, and that the impairment of this pathway may drive the pathogenesis in sporadic AD by disrupting amyloid clearance [81]. The role of brain pericytes in the trans-endothelial transport of Aβ proteins and their clearance is unclear and will require further investigation. Nonetheless, disruptions in BBB homeostasis that impact Aβ degradation and/or clearance via the NVU can result in the long-term accumulation of Aβ proteins, subsequently contributing to neurodegeneration and AD progression. Figure 3 shows a hypothetical model of how Aβ protein accumulation affects the function of brain pericytes in the NVU. The effects include pericyte loss, pericyte toxicity, induction of ROS, and capillary constriction, all contributing to neuronal toxicity and neurodegeneration in AD.

## 4. Aβ Proteins and Retinal Pericytes in the Ocular Compartment of AD Patients

The inner blood–retinal barrier (IBRB) consists of retinal microvascular endothelial cells covered with tightly associated retinal pericytes and Müller cells; together, they form the retinal vascular unit (RVU) [82,83,84,85]. Retinal and brain pericytes have similar functions—both require regulation by PDGFRβ signaling for proper pericyte recruitment and adequate barrier formation [86,87]. Both AD and age-related macular degeneration (AMD) often occur among aging populations. Human retinal pericyte exposure and response to Aβ proteins have not been extensively studied. This represents a knowledge gap that warrants further investigation.

Nonetheless, the retinal pathology observed in AD patients is similar to Aβ deposition observed in patients with AMD. Similarly, Aβ accumulation observed in the brains of AD patients also exists in the retinal compartment of both AD patients and patients with AMD [88,89]. ROS resulting from mitochondrial dysfunction are also present in both AD patients and patients with AMD. Recent studies demonstrated that long-term treatment with mitochondria-targeted antioxidant SkQ1 suppressed the development of AMD-like pathology in senescence-accelerated OXYS rats by reducing the level of Aβ proteins and suppressing the activity of mTOR in the retina [90,91,92]. Findings from these studies revealed a connection between AD with AMD and may provide insights into the development of novel strategies for treating AD. Aβ plaques also exist in the brains and retinas of animal models of AD; in fact, the accumulation of Aβ proteins can occur in the retina before the brain [93,94,95,96,97]. As a result, animals with retinal Aβ accumulation experience significant changes in vision [98,99]. This is because Aβ toxicity results in the upregulation of MCP-1 and apoptotic markers in the ganglion cell layer [100,101,102], leading to microglia infiltration and astrogliosis in the retina.

In a study by den Haan et al. that examined the presence of Aβ, pTau, and APP in the post-mortem retinas of AD patients and controls by immunohistochemistry, immunostaining for Aβ and APP yielded positive results in ganglion cells, amacrine cells, horizontal cells and Müller cells in both controls and AD cases [103]. Aβ plaque development in the retinal tissue of AD patients has been controversial because the ocular pathologies observed can also be due to other confounding factors such as ocular hypertension, multiple sclerosis, and Parkinson’s disease [88,93]. In other words, it is currently not possible to directly link Aβ deposition in the retina to AD manifestation. Nonetheless, a study by Koronyo-Hamaoui et al. revealed the presence of Aβ plaques in post-mortem retinal tissue, and that the formation of these plaques preceded plaque formation in the brain that correlated with AD progression [93].

Modern photonic imaging systems such as optical coherence tomography (OCT) could provide a non-invasive means for visualizing retinal changes in AD that occur before degenerative changes in the brain. The development of these ocular amyloid biomarkers for AMD could potentially provide unique diagnostic tools for AD especially at early stages, for monitoring AD disease progression, and for gauging the effects of novel treatment strategies. In a study by Ferrari et al. using OCT, they observed retinal neuroaxonal thinning in frontotemporal dementia, as seen in AD [104]. Changes in retinal nerve fiber layer (RNFL) thickness is thought to occur because of retrograde degeneration of the retinal ganglion cell axons, and these changes have been suggested to occur before memory is affected [105]. OCT appears to be a promising method to aid in the diagnosis of various neurodegenerative disorders, including AD. However, the clinical severity of AD and the overall RNFL thickness is not well established and will require further investigations [106]. Most recently, Szegedi et al. have shown anatomical and functional changes in the retina in patients with AD and mild cognitive impairment using OCT [107]. They found that, in patients with MCI and AD, arteriovenous differences in oxygen saturation, retinal blood flow and arterial vessel diameter was reduced, which suggests alterations in retinal oxygen metabolism in patients with AD [107]. In addition, in vivo two-photon microscopy (TPM) in mouse models of AD have revolutionized visual imaging in real time in the intact brain, such as for the examination of individual Aβ plaques, and the effects of anti-amyloid therapies on plaque formation and growth [108]. TPM combined with fluorescent calcium indicators revealed hyperactivity-associated Aβ deposition. This observation was found to be associated with cortical and hippocampal neurons that were revealed as a primary neuronal impairment [108].

## 5. Strategies to Replace Pericytes in the CNS or Protect Them from the Effects of Aβ

It would be interesting to determine if gene expression profiles of brain pericytes differ from those of pericytes in other microvascular compartments (eye, placenta, and kidney) upon exposure to Aβ proteins. For instance, some pericytes may be unresponsive to the Aβ-mediated generation of ROS and induction of endothelin-1. The analysis of gene expression profiles of different pericytes upon exposure to Aβ proteins could help researchers identify pericyte populations that are unresponsive to Aβ-mediated capillary constriction. This discovery would support the transfer of Aβ-nonresponsive pericytes from one microvascular compartment to another as a therapeutic intervention. To this end, Tachibana et al. examined the effects of pericytes on Aβ metabolism using mouse mesenchymal stem cells that had been differentiated into pericytes and stereotaxically injected into the brains of APP/PS1 mice, an animal model for amyloid AD [109]. Upon implantation, the mice exhibited increased brain microcirculation, reduced levels of insoluble hippocampal Aβ_40_ and Aβ_42_ in the pericyte-injected hemisphere and reduced hippocampal Aβ deposition [109]. This study suggests that cell-based therapies involving pericyte implantation may provide a novel approach to treating or limiting the progression of AD.

Pericyte replacement is currently being investigated as a treatment option for non-proliferative diabetic retinopathy (NPDR). This condition presents in diabetic patients when their retinas exhibit microaneurysms, microhemorrhages, nerve fiber infarcts (also known as cotton wool spots), retinal edema, and intraretinal vascular abnormalities [110]. NPDR is also characterized by the loss of both pericytes and endothelial cells, resulting in a reduced number of functional blood vessels and the disruption of the BBB [110]. The treatment replaces pericytes lost in the early stages of NPDR with adult MSCs from the bone marrow that can differentiate into pericytes under certain conditions [111]. In this regard, the RETICELL clinical trial examined the intravitreal injection of adult bone marrow MSCs into patients with retinitis pigmentosa in an attempt to restore ocular function [112]. The fact that this treatment could improve the patients’ quality of life, even temporarily, demonstrates the possibility of pericyte replacement as a viable treatment option [112]. Another strategy is to protect pericyte loss by preventing or limiting the generation of ROS that contribute to pericyte loss. Pigment epithelium-derived factor (PEDF), secreted by primary human adipocytes, is a potent antioxidant that is neurotrophic and antiangiogenic [113,114]. A study of recombinant PEDF applied topically to the eyes of diabetic Akita mice showed diminished pericyte death, vascular leakage, and inflammation in the retina [115]. Other potential therapeutic strategies to prevent pericyte loss include blocking the voltage-gated Ca2+ channels on the pericytes, a method that has been shown to slow ischemia-mediated capillary constriction by the pericytes [116]. In addition, thalidomide, despite its toxicity, has been shown to induce pericyte proliferation, recruit pericytes to capillaries, and induce vessel maturation by increasing the levels of PDGF-B homodimers in endothelial cells [117,118]. Additionally, thalidomide has been shown to reduce BBB leakiness in an AD animal model [119]. Moreover, thalidomide has been patented for use to prevent the loss of BBB function after radiation therapy [120].

## 6. Conclusions

Pericytes are multipotent cells of the vascular system. The interaction between endothelial cells and pericytes is essential for proper microvascular formation, development, stabilization, and maintenance. These cells also influence the chemical composition of the surrounding microenvironment to protect endothelial cells from potential harm. The dysregulation or loss of pericyte function can result in microvascular instability and pathological consequences.

Since pericyte death reliably indicates mild cognitive impairments, it is likely both a symptom and a potential cause of AD. Hypoxia affects mechanisms involving APP. In doing so, it leads to the increased production of β- and γ-secretase, and, subsequently, increased Aβ production. Therefore, pericyte loss due to Aβ toxicity may be an early event in AD progression, as pericyte loss reduces CBF and thus creates hypoxic areas throughout the brain. In other words, pericyte loss contributes to neuronal dysfunction and neurodegeneration over time. Drugs targeting pathophysiological mechanisms including Aβ or tau protein metabolism, mitochondrial dysfunction, oxidative stress, and inflammation have been unsuccessful in treating AD. This strongly supports the notion that AD is a multifactorial disease that likely involves changes in the brain microvasculature and pericytes. These changes suggest that therapies aimed at maintaining normal pericyte function may preserve neuronal function by preventing the decrease in CBF and the loss of BBB function. Therefore, therapies that prevent capillary constriction by pericytes and pericyte death may promote the restoration of CBF and the maintenance of BBB function after ischemia.

Both pericyte loss and dysfunction are involved in the development of fibrosis, diabetic retinopathy, ischemic organ failure, pulmonary hypertension, Alzheimer’s disease, Parkinson’s disease, tumor growth, and metastasis. Animal models designed to develop therapies for pericyte loss or dysfunction have limitations. Mouse models often fail to reproduce the anatomical complexity of the human brain and its vascular systems that can contribute to misleading results in clinical trials. The phenotypic differences among pericyte populations and how these populations function requires further investigation. The lack of standardized methods and procedures for pericyte isolation and characterization limit their use in clinical trials [121]. Clinically, pericytes have been limited to regenerative medicine and mainly used for the repopulation of vascular grafts [122]. The limitations for pericyte use in clinical trials can be addressed in the future by the identification of tissue-specific phenotypes and functions, as well as the standardization of the isolation and purification of pericytes that can be reproducible and established in a clinical setting for pericyte-based therapies.
